# Tributary Inflows to a Regulated River Influence Bacterial Communities and Increase Bacterial Carbon Assimilation

**DOI:** 10.1007/s00248-023-02271-1

**Published:** 2023-07-22

**Authors:** Lauren O’Brien, Nachshon Siboni, Justin R. Seymour, Matthew Balzer, Simon Mitrovic

**Affiliations:** 1 School of Life Sciences, University of Technology Sydney, Ultimo, NSW Australia; 2 Climate Change Cluster, University of Technology Sydney, Ultimo, NSW Australia

**Keywords:** Tributaries, Bacterial carbon production, 16S DNA, River metabolism, Bacterial community composition

## Abstract

Inflows from unregulated tributaries change the physical, chemical, and biotic conditions in receiving regulated rivers, impacting microbial community structure and metabolic function. Understanding how tributary inflows affect bacterial carbon production (BCP) is integral to understanding energy transfer in riverine ecosystems. To investigate the role of tributary inflows on bacterial community composition and BCP, a ~90th percentile natural flow event was sampled over 5 days along the Lachlan River and its tributaries within the Murray-Darling Basin of eastern Australia. Increased tributary inflows after rainfall corresponded with a significantly different and more diverse bacterial community in the regulated mainstem. The major contributor to this difference was an increase in relative abundance of bacterial groups with a potential metabolic preference for humic substances (Burkholderiaceae *Polynucleobacter,* Alcaligenaceae *GKS98 freshwater group*, Saccharimonadia) and a significant decrease in Spirosomaceae *Pseudarcicella*, known to metabolise algal exudates. Increases in orthophosphate and river discharge explained 31% of community change, suggesting a combination of resource delivery and microbial community coalescence as major drivers. BCP initially decreased significantly with tributary inflows, but the total load of carbon assimilated by bacteria increased by up to 20 times with flow due to increased water volume. The significant drivers of BCP were dissolved organic carbon, water temperature, and conductivity. Notably, BCP was not correlated with bacterial diversity or community composition. Tributary inflows were shown to alter mainstem bacterial community structure and metabolic function to take advantage of fresh terrestrial dissolved organic material, resulting in substantial changes to riverine carbon assimilation over small times scales.

**Supplementary Information:**

The online version contains supplementary material available at 10.1007/s00248-023-02271-1.

## Introduction

River regulation interrupts the longitudinal flow of biota and resources from river headwaters and the surrounding catchment, which can subsequently impact riverine microbial community structure and function [1–3]. Dams and weirs create lentic environments along river channels where thermal stratification can create conditions that favour algal blooms or anoxia and nutrient release from sediments in the hypolimnion, which fuels primary production downstream and shifts rivers into net autotrophy [4–6]. River regulation also reduces connectivity to the catchment by reducing the frequency and magnitude of average annual flows and floods downstream of the dam, as well as changing the seasonal distribution of flows through the decoupling of rainfall and flows [7–9]. These altered flow regimes diminish the amount of dissolved organic carbon (DOC) exported from the floodplain, with models predicting reductions in annual DOC export of up to two-thirds of natural levels under regulated scenarios [10, 11].

Tributaries of major rivers are often unregulated or less regulated than the river mainstem, so their flow regimes and flow-related resource pulses can help to restore degraded river mainstems to a more natural state [12–14]. Tributary inflows alter the physical, chemical, and biological conditions in river mainstems through temperature shifts and the import of fresh dissolved organic matter (DOM), nutrients, sediment, and biota from tributary headwaters and the greater catchment [12, 14–16].

The changed physical and chemical environment in regulated rivers during tributary inflows causes a corresponding shift in planktonic microbial community composition and metabolic function towards one more representative of surrounding floodplain habitats [3]. Bacterioplankton communities shift almost immediately during tributary inflow events due to mobilisation of bacteria from catchment soil, the hyporheic zone, and upstream aquatic microhabitats [3, 17, 18]. These disparate microbial communities can come together to form a new community with emergent properties distinct from its components in a process known as microbial community coalescence [19]. The altered bacterial community structure and increased availability of complex terrestrial DOM can drive increased pelagic bacterial carbon production (BCP), which captures DOC and nutrients into bacterial biomass making them biologically available to higher trophic levels via the microbial loop [20–23]. Community mixing effects are typically short lived (~1–2 weeks, depending on catchment complexity), and over the following days and weeks, processes such as environmental filtering, competition, and predation become the dominant structuring forces of riverine bacterioplankton communities [24, 25]. However, frequent tributary inflows may lead to altered bacterial communities in mainstem rivers over time, and even transient resource pulses can continue to subsidise the food web for months after microbial assimilation [3, 26].

Linking tributary inflow characteristics with their impact on mainstem bacterioplankton communities and subsequent changes in BCP and microbial carbon assimilation is integral to modelling overall energy transfer in riverine ecosystems [27]. However, it is not well understood how effective these inflows are at supporting instream BCP during the crucial initial stages of a flow-based resource pulse. This is particularly so in Australia’s highly regulated Murray-Darling Basin, where nearly 70% of mean annual inflow is captured through dams and irrigation pumping [28]. For example, an 18-month seasonal study of BCP on the Murray River recorded the highest planktonic BCP rates (> 60 μg C L^−1^ h^−1^) directly below an unregulated tributary during an inflow event, but relationships with tributary inflow characteristics could not be identified due to infrequent sampling [29]. In addition, responses to resource pulses are often studied only during extreme hydrological events (i.e. large-scale flooding, drought) with comparably extreme and transient impacts on river ecology [30–32]. Moderate flow pulses (~90–95th percentile flows) are far more frequent in these systems and hence may be more important to the structure and function of microbial communities than larger, more infrequent events.

The aim of this study was to examine the immediate impact of moderate-sized inflows from tributaries on heterotrophic BCP and bacterioplankton community structure in a major regulated river and investigate the possible mechanisms behind any changes observed. Water column DOC, nutrients, physico-chemical parameters, BCP, and bacterial DNA were sampled daily on the Lachlan River and its tributaries in central New South Wales Australia before and during a four-day rainfall event of ~60 mm. It was hypothesised that tributary inflows would alter environmental conditions and bacterial community composition in the river mainstem, which would increase BCP per litre, thus increasing the total load of carbon assimilated by riverine bacteria.

## Methods

### Study Sites

The Lachlan River is an inland floodplain river and major tributary of the Murray River in central New South Wales, Australia. This region experiences an average annual rainfall of 600 mm. Flows of the Lachlan River are regulated by Wyangala Dam (1217 GL, catchment size 8300 km^2^), with the river having a largely agricultural catchment, consisting mainly of grazing and dryland cropping [33]. Downstream of Wyangala Dam, the Lachlan River is fed by several unregulated (or only moderately regulated) tributaries. Of importance to this study are Boorowa River, entering the river 18 km downstream of the dam wall with a catchment size of 1500 km^2^, and Belubula River entering the river 102 km downstream of the dam wall with a catchment size of 1600 km^2^. The Belubula River is regulated by a small dam (Carcoar Dam 36 GL, catchment size 230 km^2^) ~130 km before its confluence with the Lachlan River but maintains an almost natural hydrograph at the confluence [34].

**Fig. 1:**
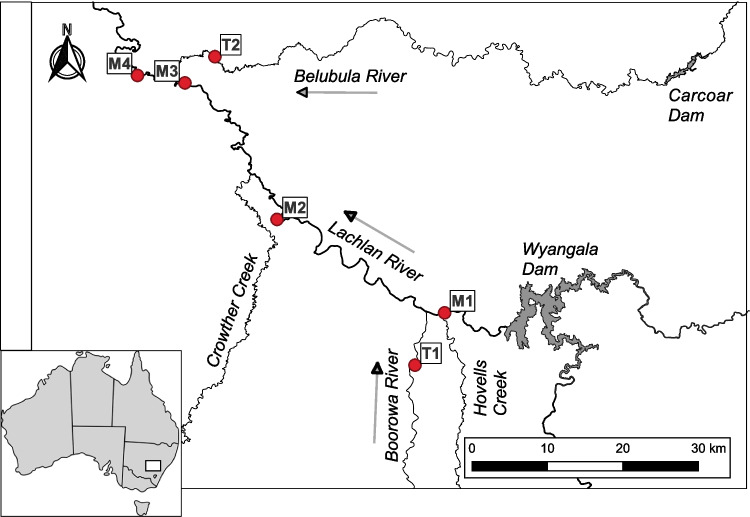
Location of study sites on the Lachlan, Boorowa, and Belubula Rivers in central NSW Australia. Sites are denoted with dots. Arrows indicate the direction of river flow

Samples were collected from six sites along the mid-reaches of the Lachlan River and two adjoining tributaries (Boorowa and Belubula Rivers) (Fig. 1). Four sites were spaced along the Lachlan River mainstem to capture instream conditions upstream and downstream of major tributary confluences. Two smaller tributaries, Crowther and Hovells Creek, were not sampled due to insufficient water depth at base flow (Crowther ~0.17 m^3^ s^−1^, Hovells ~0.06 m^3^ s^−1^).

### Sample Collection and Analysis

Samples were collected as close to the same time of day as possible over 5 days, before and during a moderate natural flow event over the week of 23–27 October 2020. Sites were prioritised for sampling each morning, based on current hydrographs from local gauges. At each site, 10 L of water was collected from 20 to 30 cm below the surface. Because ecology at the microbial level is heterogenous at a small spatial scale [35], a composite sample was made up of six combined ~1.5 L subsamples and combined in an acid-rinsed (10% HCl) polyethylene bottle.

For DOC, nitrogen oxides (NOx), ammonia, and orthophosphate (RP) analyses, 200 mL of river water was filtered through pre-flushed 0.45 μm PTFE filters in triplicate, with filtered water then frozen until analysis. DOC was determined using a Shimadzu TOC-L analyser and NOx, ammonia, and RP were determined by flow injection analysis on a Lachat Quikchem 8500. Blanks, certified reference materials, spiked matrices, and analytical duplicates were used for quality control. Water column temperature, conductivity, dissolved oxygen, and pH were sampled with a Hydrolab Surveyor and MS5 Minisonde.

### Bacterial Carbon Production

Bacterial carbon production (BCP) was determined using an amended version of the ^3^H-leucine incorporation method of Smith and Azam [36]. Optimum ^3^H-leucine concentration was determined by creating a saturation curve (15, 30, 60, 120 nM) with composite samples from the sample sites 2 weeks before the flow event as in Buesing and Gessner [37]. Sample water was filtered to 2.7 μm through a sample-flushed glass fibre filter to remove bacterial predators [38]. Triplicate 1 mL aliquots of sample water were then dark incubated in 2 mL sterile-screw cap centrifuge tubes with o-rings with ^3^H-leucine (120 nM 1:5 ^3^H-leucine:unlabelled leucine). Incubations were conducted at the field sites in an insulated container at ~18–20 °C for 1 h before incubations were terminated with cold 5% w/v trichloroacetic acid (TCA). One killed control, pre-treated with cold TCA, was included for every 12 replicates. Samples were then centrifuged, the supernatant was siphoned off, and the pellets were rinsed with ice-cold 5% TCA twice before being combined with Ultima Gold II scintillation fluid and radioactivity measured using a PerkinElmer Tri-Carb 2810 TR scintillation analyser. BCP was calculated from bacterial protein production using a conversion factor of 0.86 [39] with an internal isotope dilution factor of one assumed due to the high concentration of ^3^H-leucine used [40]. BCP measurements are communicated as either volumetric BCP (BCP_vol_ μg C L^−1^ h^−1^) or total load of BCP in the water column during the hour of sampling (BCP_load_ g C). BCP_load_ was calculated as BCP_vol_ multiplied by hourly discharge at the time of sampling. To determine if BCP was nutrient limited, an amendment of Lindstrom’s [41] growth media L16 (containing nitrate, phosphate, and micro-elements) was made without carbon and added to replicates of ^3^H-leucine incubations to determine what the maximum BCP rate was when not nutrient limited [42].

### 16S rRNA Gene Sequencing

Selected days at sites M2, M3, M4, T1, and T2 were sequenced to capture important changes in the microbiome based on preliminary BCP results. Site M1 was not sequenced due to negligible change in discharge or water source. Sample water was filtered through 47 mm, 0.22 μm PVDF membrane filters using a peristaltic pump (100 rpm). Filters were snap frozen in liquid nitrogen and stored at -80 °C until DNA was extracted using Qiagen DNeasy PowerWater Kit, according to the manufacturer’s instructions. Negative controls were extracted from field and lab blank filters.

DNA was amplified using the 16S rRNA primers; Bakt_341F and Bakt_805R, which amplify the V3–V4 region [43], with the following cycling conditions: 95 °C for 3 min followed by 25 cycles of: 95 °C for 30 s, 55 °C for 30 s, 72 °C for 30 s, and then 72 °C for 5 min with a final hold at 4 °C [44]. Amplicons, including negative controls, were checked on agarose gel to ensure the integrity of the filter and DNA extraction. Then sample amplicons were sequenced on the Illumina Miseq platform (2 × 300 bp) following the manufacturer’s guidelines at the Australian Genome Research Facility, Melbourne (AGRF). Raw data files in FASTQ format were deposited in the NCBI Sequence Read Archive (SRA) under Bioproject number PRJNA943514.

Raw demultiplexed 16S rRNA gene data were processed using the Quantitative Insights into Microbial Ecology (QIIME 2 version 2020.6.0) pipeline [45]. Briefly, paired-ended 16S DNA sequences were imported, then trimmed and denoised using DADA2 version 2020.6.0, which also removes chimeras [46]. The classify-sklearn qiime feature classifier was used to assign taxonomy against the Silva v138 database [47] at the amplicon sequence variant (ASV) level. The dataset was further cleaned by removing ASVs with less than 50 reads (0.01% of the total reads) and those identified as chloroplasts, mitochondria, or as unassigned sequences. Cleaned data were then rarefied at 3530 reads per sample (Supplementary figure S1).

### Statistical Analysis

All statistical analysis was carried out in R version 4.1.3 [48]. Hourly flow rates at each site were estimated by time-shifting data from the nearest discharge gauge [34] to accommodate estimated water travel time to/from the gauge. Water transit times were calculated based on the time difference of peaks in discharge at consecutive gauges and equated to river distance measured in QGIS 3.24.2 [49]. Water velocity was estimated at 0.49 m s^−1^. Estimates may vary in accuracy due to changes in water velocity with magnitude of discharge but provide a more accurate estimate than using unadjusted gauge readings.

Relationships were explored between hourly flow rate, basal resources, bacterial community diversity, bacterial taxa relative abundance, and BCP_vol_ using Pearson’s correlation tests. For statistical comparison, hourly flow rate was categorised into three groups: base flow, small inflow, and event flow. These categories were based on hydrographs and field observations of rainfall and changes in river height. Briefly, small inflows were classed as when flows more than doubled from base flow, and event flow was classed as flows over 2.9 m^3^ s^−1^. Differences in BCP and basal resources between flow categories and sites were compared with ANOVA or *t*-tests. Where the assumption of equal variance was not satisfied, Kruskal-Wallis or Wilcoxon rank sum tests were used as non-parametric alternatives. Drivers of BCP_vol_ were modelled using stepwise multiple regression in the *MASS* package, and best models were validated by running a simulated model fit and selecting the best RMSE and R2 [50, 51]. To understand how much additional carbon was captured by the system in total during a flow event, BCP_load_ was calculated for the hour during which sampling occurred only, with the assumption that BCP_vol_ rates remained relatively stable over that hour. Loads were not modelled over the entire study period due to uncertainty in the ecological significance of the correlation between discharge and BCP_vol_ rates and the high variability in BCP_vol_ rates over small time periods [35].

Bacteria community composition was SIMPROF clustered with no a priori assumptions using *clustsig* to determine whether spatial or temporal factors were more important in the similarity between communities [52]. Differences in community composition between flow categories were assessed by ANOSIM using Bray-Curtis dissimilarity, with multivariate dispersion first tested using betadisper in *vegan* version 2.6.4 [53]. ASVs contributing the most to these differences were then identified with SIMPER. A Bonferroni correction was applied for pairwise ANOSIM comparisons. Shannon diversity was also calculated using *vegan* and significance tested using Kruskal-Wallis and Dunn’s tests. Differences in the relative abundance of taxa were tested with ANOVA. Environmental drivers of bacterial community composition were modelled with nMDS and stepwise dbRDA using *vegan* with best model fit assessed by permutation. Correlations between the 50 most abundant ASVs and environmental variables were visualised using network analysis. Pearson’s correlations were first generated using MICtools and the network was visualised in Cytoscape version 3.9.1 [54].

Figures were generated using *ggplot2*, and maps created in QGIS [55].

## Results

### Hydrology and Resource Mobilisation

**Fig. 2:**
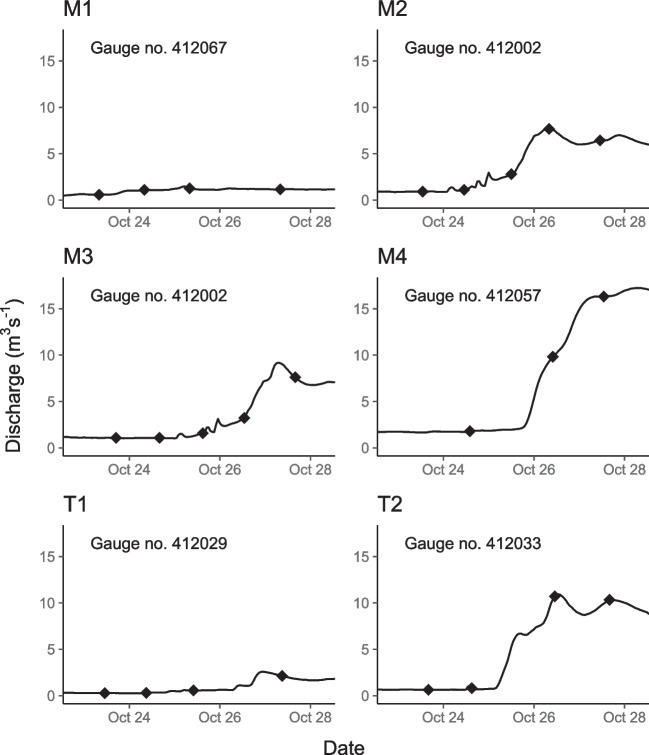
Hydrographs for study sites from the Lachlan River catchment during 23–28 October 2020. Sample times are marked with (◆). Water NSW gauge number used to calculate discharge is listed for each site [34]

All rivers in the local catchment had been at base flow for ~4 months prior to sampling. During the study period, rainfall caused a local flow pulse down the Lachlan River mainstem and a substantial flow pulse down the T2 tributary (mean daily discharge 9.5 m^3^ s^−1^, 92nd percentile). A minor flow pulse also occurred on the T1 (2.2 m^3^ s^−1^, 82nd percentile), Hovells Creek (2.5 m^3^ s^−1^, 93^rd^ percentile), and Crowther Creek (0.81 m^3^ s^−1^, 84^th^ percentile) tributaries (Fig. 2). DOC concentrations ranged between 7 and 13 mg L^−1^ and were significantly higher during small inflows than base flows at all sites except M1 (all except M1 *χ*^2^ = 12.9, *p* = 0.002; M1 only *t* = 1.69, *p* = 0.14, Table 1). At M1, concentrations were significantly higher than other sites throughout the study period despite little change in discharge at this site (12.1 ± 1.2 mg L^−1^, *F*_(5,44)_ = 5.2, *p* = 0.001, Table 1). RP concentrations ranged from 1 to 36 μg L^−1^ and were up to 7 times higher during event flow within tributaries and significantly elevated at sites directly downstream of tributary confluences during event flow (all except M1 *χ*^2^ = 11.6, *p* = 0.003, Table 1). NOx and ammonia significantly increased by up to 8-fold during event inflows at T1 and all other mainstem sites, but NOx did not increase with flow at T2 (NOx all except T2 *χ*^2^ = 22.8, *p* < 0.001; T2 only *W* = 8, *p* = 1, Table 1). Temperature dropped significantly in tributaries and at all sites below confluences over the course of the flow event from ~21 to 17 °C (all except M1 *F*_(2,60)_ = 89.4, *p* < 0.0001).

**Table 1:** Concentration of dissolved organic carbon (DOC), orthophosphate (RP), nitrogen oxides (NOx), ammonia, and total load of bacterial carbon production during the hour of sampling (BCP_load_) during a tributary-driven flow pulse at six sites in the Lachlan River catchment.

Site	Day	DOC (mg L^−1^)	RP (μg L^−1^)	NOx (μg L^−1^)	Ammonia (μg L^−1^)	BCP_load_ (g C)
Mainstem sites						
M1	1	13.2 ± 1.3	5.27 ± 0.45	94.0 ± 2.8	147 ± 4.2	10.1 ± 0.20
	2	10.8 ± 0.35	6.17 ± 0.76	174 ± 1.4	149 ± 0.71	11.8 ± 0.21
	3	11.6 ± 0.11	12.1 ± 0.42	294 ± 11	116 ± 19	11.1 ± 0.29
	5	12.8 ± 1.0	8.79 ± 0.057	411 ± 2.1	94.2 ± 8.3	8.37 ± 0.22
M2	1	8.33 ± 0.12	1.76 ± 0.42	2.45 ± 1.4	14.8 ± 1.2	13.4 ± 0.090
	2	7.56 ± 0.19	2.7 ± 1.5	97.1 ± 3.5	17.9 ± 1.1	12.2 ± 0.37
	3	8.29 ± 0.63	2.15 ± 0.014	122 ± 1.4	50.0 ± 2.5	21.2 ± 0.69
	4	10.7 ± 0.20	11.1 ± 0.64	373 ± 1.4	99.4 ± 0.92	54.1 ± 2.1
	5	12.1 ± 1.8	11.9 ± 1.8	297 ± 0.71	51.4 ± 14	54.1 ± 0.79
M3	1	8.08 ± 0.12	1.11 ± 0.042	23.0 ± 22	7.27 ± 0.74	13.8 ± 0.51
	2	8.13 ± 0.32	1.33 ± 0.11	27.7 ± 0.64	17.2 ± 3.4	17.2 ± 0.070
	3	9.42 ± 2.7	2.29 ± 0.68	89.3 ± 2.3	31.3 ± 1.3	9.61 ± 0.19
	4	7.80 ± 0.020	1.86 ± 1.1	88.3 ± 1.4	17.0 ± 1.3	18.0 ± 0.51
	5	8.94 ± 0.065	4.66 ± 0.54	429 ± 9.2	38.3 ± 1.4	70.3 ± 1.0
M4	2	9.22 ± 2.3	2.97 ± 0.49	109 ± 2.1	19.1 ± 0.071	17.6 ± 0.13
	4	7.68 ± 0.57	7.66 ± 2.0	459 ± 21	51.6 ± 9.6	39.1 ± 1.0
	5	8.79 ± 0.0030	11.3 ± 2.4	299 ± 4.2	43.4 ± 2.2	118 ± 2.2
Tributary sites						
T1	1	7.64 ± 0.23	3.67 ± 0.53	3.88 ± 0.85	7.25 ± 0.092	3.39 ± 0.052
	2	8.33 ± 0.17	5.68 ± 1.4	49.0 ± 1.8	6.05 ± 80	6.84 ± 0.16
	3	12.2 ± 0.32	36.3 ± 0.49	318 ± 1.4	36.8 ± 2.3	7.39 ± 0.23
	5	9.45 ± 0.24	8.82 ± 0.16	198 ± 61	19.3 ± 0.14	16.7 ± 0.42
T2	1	7.14 ± 0.61	5.86 ± 1.7	396 ± 14	13.7 ± 0.49	3.79 ± 0.073
	2	7.08 ± 0.63	4.76 ± 1.0	358 ± 1.4	25.0 ± 5.9	7.59 ± 0.046
	4	9.34 ± 0.15	19.6 ± 0.35	381 ± 2.8	36.3 ± 2.2	44.0 ± 0.67
	5	12.1 ± 1.7	19.6 ± 0.49	283 ± 1.4	28.8 ± 5.8	75.7 ± 0.85

### Bacterial Carbon Production

**Fig. 3:**
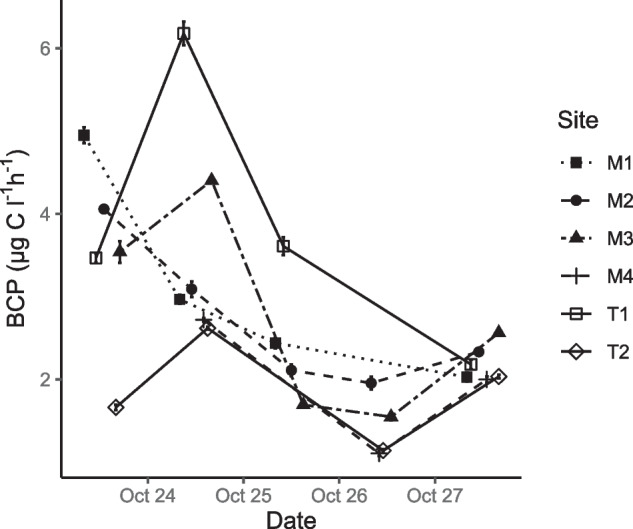
Change in rate of volumetric bacterial carbon production (BCP) during a tributary-driven flow pulse at six sites in the Lachlan River catchment

The rate of BCP_vol_ (μg C L^−1^ h^−1^) significantly decreased during the first 2 days of increased flow, then began to recover significantly at most sites on the final day of sampling (*F*_(4,70)_ = 24.3, *p* < 0.0001), except for M1 and T1 which continued to drop (Fig. 3). The initial rate of BCP_vol_ at base flow was disparate between sites, with BCP_vol_ being statistically highest at M1 and lowest at T2 (*F*_(5,12)_ = 86.3, *p* < 0.0001), but rates became more similar across sites on the final day of sampling (BCP_vol_ start range = 1.7–4.9, end range = 2.0– 2.6 μg C L^−1^ h^−1^, Fig. 3). There was a significant increase in BCP_vol_ during base flow at T1, T2, and M3 on the second day of sampling that corresponded with observations of initial direct rainfall (not sufficient magnitude to register on discharge gauge) and increased river channel wetting (*F*_(3,8)_ **≥** 939, *p* < 0.0001, Fig. 3). Stepwise multiple linear regression identified significant positive drivers of *log10* BCP_vol_ as DOC, temperature, and conductivity (adj *R*^2^ = 0.56, *F*_(3,21)_ = 11.12, *p* = 0.0001). No association could be found between BCP_vol_ and bacterial community parameters or relative abundance of bacterial taxa. There was no difference in BCP between L16 growth media amended incubations and controls (Supplementary table S2).

Despite BCP_vol_ rates per litre dropping during the flow event, the total load of carbon assimilated by riverine bacteria during the hour of sampling (BCP_load_) increased significantly by 4–20 times during the flow event due to the increased volume of water in the river channel, except for at M1 due to little change in discharge at this site (all except M1 *F*_(2,18)_ = 7.5, *p* = 0.004; M1 only *t* = −0.13, *p* = 0.91; Table 1).

### Bacterial Community Relative Abundance and Composition

**Fig. 4:**
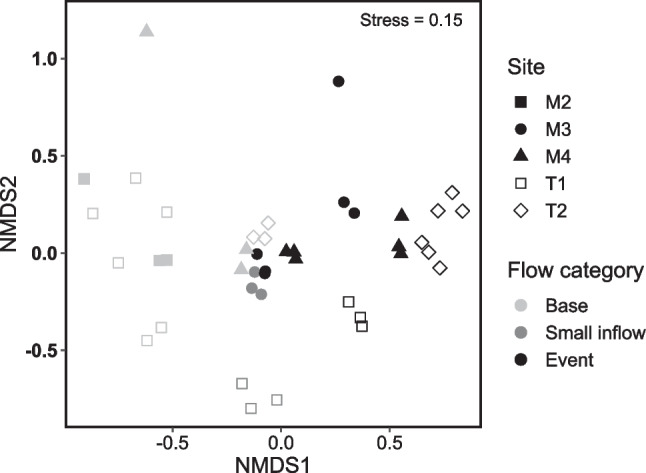
nMDS ordination based on Bray-Curtis dissimilarity showing bacterial community composition during a tributary-driven flow pulse at five sites in the Lachlan River catchment

**Fig. 5:**
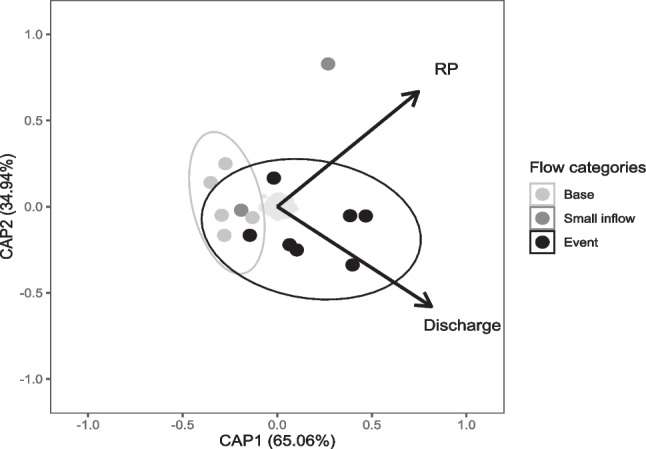
Distance-based redundancy analysis (dbRDA) using Bray-Curtis dissimilarity showing influence of environmental variables on bacterial community composition during a tributary-driven flow pulse at five sites in the Lachlan River catchment. Only significant variables orthophosphate (RP) and discharge (hourly discharge) (*p* = 0.001) are shown as vectors, and ellipses represent 95% CI of the mean of each flow category

Shannon diversity was significantly higher during the flow event compared to base and small inflows at all sites (*χ*^2^ = 18.19, *p* = 0.0001, Dunn’s *p* < 0.008). nMDS ordination of bacterial ASVs showed a clear grouping of base flow and event flow communities (Fig. 4). Event flow communities were significantly different to those during base and small inflows (ANOSIM overall *R* = 0.49, *p* = 0.0001, event-base *R* = 0.32, *p* = 0.0001, event-small inflow *R* = 0.45, *p* = 0.0001), and across the study period the community at T2 was significantly different to that at T1 and all other upstream sites (ANOSIM overall *R* = 0.26, *p* = 0.0001, T2-upstream *R* > 0.42, *p* < 0.005). SIMPROF clustering indicated the closest level of bacterial community similarity was between each tributary and the site directly downstream of their confluence, usually on the same day (Supplementary figure S3). Orthophosphate concentration and hourly discharge explained 31% of the variation in bacterial community composition across all sites, with communities clearly grouped by flow category in a distance-based redundancy analysis ordination (*F*_(2,11)_ = 2.43, *p* = 0.001, Fig. 5).

The ASVs contributing most to the difference in bacterial communities between flow categories belonged to the genera Spirosomaceae (*Pseudarcicella)*, Burkholderiaceae (*Polynucleobacter)*, and Alcaligenaceae (*GKS98 freshwater group)* (Supplementary table S4). The top contributing ASVs in these genera were all less abundant during the flow event than base or small inflows, with the exception of one *Polynucleobacter* ASV and two *Pseudarcicella* ASVs, which were more abundant during the flow event (Supplementary table S4). ASVs within the same three bacterial genera contributed most to the difference between tributary communities, with all ASVs more abundant at T2 than T1 (Supplementary table S5).

**Fig. 6:**
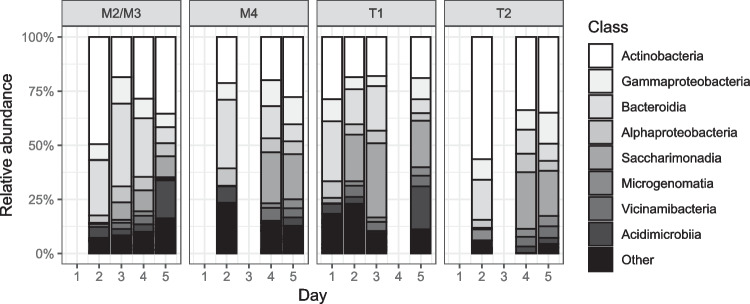
Mean relative abundance of bacterial classes throughout the study period at tributary sites T1, T2, and mainstem sites M2, M3, and M4 in the Lachlan River catchment NSW. Data from sites M2 and M3 has been pooled as 16S rRNA data was only available from site M2 on day 2 (base flow) and M3 on days 3–5 (event flow)

**Fig. 7:**
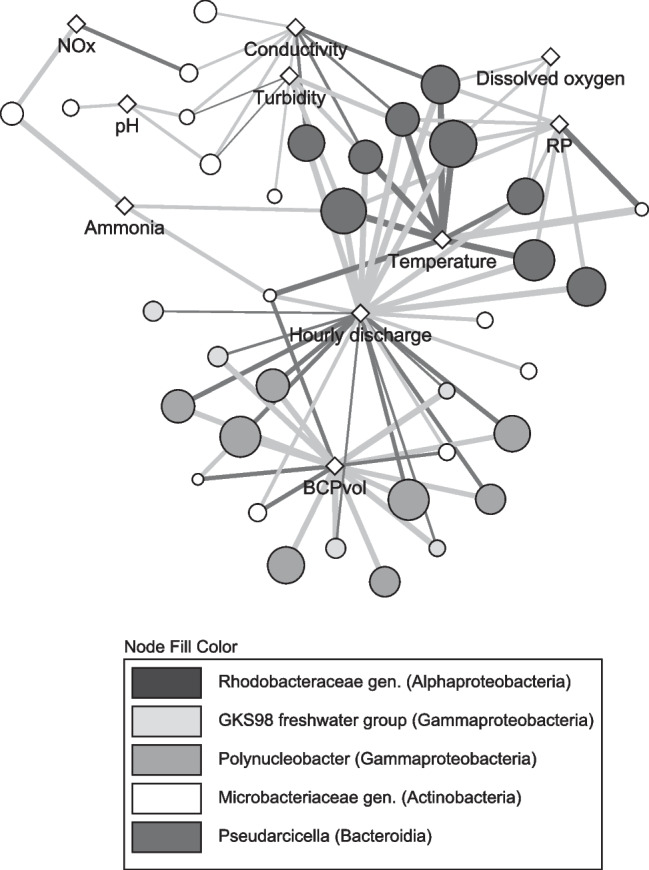
Network analysis of Pearson’s correlations between the 50 most abundant bacterial amplicon sequencing variants (ASVs) and environmental variables during a tributary-driven flow pulse in the Lachlan River catchment NSW. Dark grey edges represent positive correlations, and light grey edges represent negative correlations with width of the line representing the strength of the relationship (absolute *r* range = 0.12–0.76). Node fill shade denotes bacterial genus of the ASV, and node size represents relative abundance within the top 50 ASVs (range 1–4%). Only significant correlations are presented

At the genus level, mean relative abundance of *Pseudarcicella* decreased significantly from 12% ± 5 to 2% ± 0.8 during event flow (*F*_(4,37)_ = 5.8, *p* = 0.001), where there was a significant increase in abundance of *GKS98* and *Polynucleobacter* at the onset of event flow that was sustained in *Polynucleobacter* only (*GKS98 F*_(4,37)_ = 5.7, *p* = 0.001, *Polynucleobacter F*_(4,37)_ = 5.9, *p* = 0.001). At the class level, Saccharimonadia (phyla Patescibacteria) comprised only a small proportion of mean relative abundance at base flow (< 2%) but became one of the dominant classes during event flow at all sites (~25%), particularly at tributary sites (Fig. 6). Network analysis showed positive correlations between nearly all *Polynucleobacter* and *GKS98* ASVs and hourly discharge but negative correlations with BCP_vol_ (Fig. 7). Conversely, nearly all *Pseudarcicella* ASVs were negatively correlated with hourly discharge but positively correlated with temperature.

## Discussion

During this study, tributary inflows altered environmental conditions in the river mainstem, mainly via increased concentrations of DOC, phosphorus and nitrogen, and reduced temperatures. These environmental changes corresponded with a shift in bacterial community composition and an initial decrease in BCP_vol_. There was nevertheless an overall increase in the total amount of carbon assimilated by riverine bacteria at all sites below tributary confluences due to the increased volume of water in the river channel. These findings deliver critical new knowledge on the role that tributary inflows play in supporting BCP in a regulated river during moderate-sized flow-resource pulses.

### Tributary Inflow Impact on Bacterial Carbon Production

During base flow, there was a gradient of high to low BCP_vol_ from upstream to downstream, which is consistent with patterns observed in a longitudinal study of the Hudson River, USA [56]. This gradient could have been caused by a longitudinal reduction in the amount of labile DOM available. As water from the dam moves downstream, repeated biological and photochemical processing leaves mostly recalcitrant DOM available with few new terrestrial inputs due to a lack of lateral connectivity at low flow [57]. A previous study of the Lachlan River found no longitudinal pattern in biological oxygen demand (BOD_5_); however, this study was conducted in the lower reaches of the river where the system becomes distributary and flow is regularly interrupted by weirs [32]. Interestingly, measures of bacterial metabolism did not respond to nutrient amendments of nitrate and phosphate in the aforementioned or current study, suggesting BCP in the Lachlan River may not generally be limited by inorganic nutrients.

Tributary inflows did not initially appear to stimulate or suppress BCP_vol_ rates at mainstem sites below confluences, even though BCP_vol_ rates at T1 were much higher than those in the mainstem and those at T2 were lower. However, BCP_vol_ rates eventually began to increase at all study sites downstream of tributary inflows on day 5. As diverse lotic bacterial communities can adapt quickly to changed conditions in the regulated mainstem [25], it is possible that the delayed response time in BCP_vol_ stimulation by tributary inflows was influenced by shortened mixing distances during the flow event [58]. As the flow event set in, the increased water velocity and higher proportion of water sourced from tributaries vs dam releases may have facilitated more efficient mixing of tributary-sourced DOC, nutrients, and bacteria resulting in increased BCP_vol_ rates at sites downstream of tributary confluences on day 5 [59]. This eventual increase in BCP_vol_ was not observed at M1 and T1, where rates continued to drop on the final day of sampling. This may have been an artefact of the homogenisation in bacterial community composition and BCP_vol_ rates seen across sites during the flow event, considering both sites began with higher rates of BCP_vol_ than all other sites (Fig. 3). Convergence in bacterial community composition and metabolism has been seen in other landscape-scale studies of bacterial composition during rainfall events [3, 25].

### Tributary Inflow Impacts on Bacterial Community Composition

Tributary inflows rapidly changed bacterial communities at all sites, increasing bacterial diversity and shifting the dominant taxa. Increases in discharge and orthophosphate were found to influence these changes. Bacterial communities at sites below tributary confluences more closely resembled those in the tributaries during the flow event compared to base flow, indicating that tributary inflows may also be importing bacteria to the regulated mainstem or altering conditions to support similar communities in both locations. These findings suggest that a combination of resource delivery and microbial community coalescence was responsible for the changes observed in bacterial communities during tributary inflows. The convergence in BCP_vol_ across sites observed in this study may have been an emergent property of the new microbial community formed by the coalescence of the catchment, tributary, and mainstem microbial communities [60]. Tributaries can be sources of microbial diversity in mainstem regulated rivers, even at times of base flow [61]. Provision and support of a more diverse bacterial community by tributary inflows could enhance the functional stability of the regulated mainstem ecosystem, as greater species richness and evenness is associated with improved ecosystem resilience [62, 63].

ASVs identified as Spirosomaceae, *Pseudarcicella* were dominant at most study sites during base flow, but the relative abundance of members of this group decreased substantially during event flow. *Pseudarcicella hirudinus* is known to utilise a variety of substances found in algal exudates (carbohydrates, amino acids, pyruvate) as its sole carbon source [64], and the genus has been associated with algal blooms [65] and may prefer low DOC, nutrients, and turbidity [66–68]. This suggests the changed environment during the flow event could have created hostile conditions for *Pseudarcicella*, reducing its relative abundance through a combination of dilution, competition, and community coalescence [3, 60].

As *Pseudarcicella* populations decreased in relative abundance, both the Burkholderiaceae, *Polynucleobacter*, and Alcaligenaceae *GKS98* increased significantly with tributary inflows. *Polynucleobacter* and *GKS98* are small cosmopolitan bacterial genera that mainly rely on organic acids released by the photochemical degradation of complex DOM for growth [69–72]. Increases in *Polynucleobacter* abundance of up to 50-fold have been observed in microcosms within 4 days of terrestrial DOC addition [73]. This suggests that the increase in DOC inputs from tributaries may be beneficial for these taxa’s growth.

At the class level, relative abundances of Saccharimonadia (phyla Patescibacteria) increased greatly during event flow, particularly in tributaries and mainstem sites downstream of the confluences. Patescibacteria may be considerably important in the determination of microbial metabolism during tributary inflows due to their preferential mobilisation from soils and groundwater and ability to degrade both simple and complex sources of carbon [74, 75]. They have even been indicated as potential degraders of recalcitrant hydrocarbons in studies on the microbial degradation of PET and PVC [76, 77].

The changes observed in bacterial taxa during tributary inflows from *Pseudarcicella* dominated to *Polynucleobacter* and *GKS98* dominated suggest a shift in bacterial community metabolic function across the catchment from one mostly reliant on algal exudates to one more adapted to the heterotrophic assimilation of complex terrestrial DOM. Similar responses to surface water inflows have been seen in hyporheic bacterial communities and demonstrate the metabolic adaptability of a diverse microbiome [18].

### Environmental Influences on Bacterial Carbon Production

BCP_vol_ rates in this study were positively correlated with DOC, temperature, and conductivity, similar to other global studies of riverine BCP [22, 31, 78]. Temperature positively affects BCP_vol_ and the speed at which BCP_vol_ responds to nutrient additions [22, 79]. The drop in temperature across all sites of ~4 °C during tributary inflows may have reduced BCP_vol_ and contributed to delayed response times to the increased resource loads. The change in DOC with discharge was not uniform across the catchment, with concentrations at tributary sites and M2 increasing with flow but other mainstem sites remaining at a similar concentration to base flow. However, there may have been a change in DOC composition, rather than quantity, during the flow event. The bulk of DOC in river mainstems can become recalcitrant due to repeated biological and photochemical processing; hence, bacterial communities in river mainstems are often carbon or nutrient limited [80–83]. Rainfall-driven inflows can deliver a subsidy of fresh terrestrial DOM to rivers [84]. Along with the increased bacterial diversity provided by tributary inflows, this subsidy may have unified the Lachlan River catchment’s bacterial community metabolism, creating a carbon “sponge” to take advantage of this fleeting resource. There was no detectable relationship between bacterial community composition or diversity and BCP, which suggests that changes in BCP were occurring somewhat evenly throughout the community.

Dominant small taxa (cell size < 0.22 μm) that were detected in this study, including *Polynucelobacter*, *GKS98*, and Patescibacteria, play an important role in riverine DOM metabolism and are often mobilised in large quantities from catchment soils and groundwater during flow events [24, 75, 85]. The increased importance of these bacterial taxa during tributary inflows should imply an accompanying increase in BCP; however, the relative abundance of dominant *GKS98* and *Polynucleobacter* ASVs were negatively correlated with BCP_vol_. This suggests that a related unsampled variable could be an important driver of BCP in the Lachlan River system. For example, a competitive, symbiotic, or predatory relationship between bacterial and protistan taxa can impact on bacterial production [86, 87]. Additionally, these genera’s weak utilisation of amino acids may have confounded the ^3^H-leucine incubations and erroneously caused this correlation. Both *Polynucleobacter* and *GKS98* have a high metabolic specificity for a range of carboxylic acids and acetate but only low use of typical algal exudates, including amino acids [72]. For example, in a study of diverse freshwater bodies, *Polynucleobacter* only utilised 56% of leucine in incubations [88]. It is therefore possible that some of the fluctuations measured in BCP in this study were more related to changes in community utilisation of leucine than actual changes in BCP.

Despite the overall drop in BCP_vol_ during the flow event compared to base flow, the total load of carbon assimilated by riverine bacteria (BCP_load_) increased up to 20 times during the flow event due to the larger volume of water in the river channel. This provides an important bioavailable carbon subsidy for higher trophic levels in the river food web, especially after a lengthy period of low flow with limited new resources entering the system [89, 90]. Bacterioplankton are predated upon by small zooplankton (rotifers, nauplii) and protists [21, 91]. This trophic transfer can occur extremely quickly, with heterotrophic nanoflagellates (HNFs) reaching bacterial clearance rates of > 100% day^−1^ during increased discharge [92, 93]. So, it is feasible that the increased energy captured by bacteria during a flow event could be available to higher-order consumers within 24 h [94, 95].

### Implications

How important tributary inflows are for basal food web stimulation in comparison to managed environmental water releases from dams is yet to be determined. Managed dam releases can stimulate similar increases in DOC and nutrients to natural inflows and can be managed to provide additional benefits, such as increased longitudinal connectivity during important fish breeding times [10, 96]. Although DOM molecular composition may be altered due to lentic dynamics within the dam [5, 97], collection of terrestrial materials from dry river benches with increasing distance downstream could support the food web in similar ways to natural inflows [98]. However, there are other benefits to the protection of tributary inflows that environmental flows do not provide, such as superior fish production outcomes and maintenance of “hot spots” of biotic diversity and complexity below confluences [99, 100]. The combined benefit of these outcomes along with basal food web stimulation implicates protection of tributary flows as a valuable river management tool.

## Conclusions

In this study, tributary inflows provided a significant subsidy of terrestrial carbon to the riverine food web in a regulated mainstem river. Inflows changed bacterial community composition and increased bacterial diversity in the mainstem through a combination of microbial community coalescence and environmental changes. Community changes did not appear to increase the capacity of the river microbiome to metabolise complex terrestrial DOM imported during the flow event on a per litre basis; however, the large increase in water volume in the river channel resulted in a parallel increase in riverine carbon assimilation. These findings highlight the key role tributary inflows play in maintaining microbial diversity and fuelling the food web in regulated rivers.

### Supplementary Information

ESM 1 (DOCX 1480 kb)

## Data Availability

16S rRNA gene sequencing data from the current study are available in the NCBI Sequence Read Archive (SRA) under Bioproject number PRJNA943514. All other datasets generated during the current study are available from the corresponding author on reasonable request.
