# Supplementary material for: Tributary Inflows to a Regulated River Influence Bacterial Communities and Increase Bacterial Carbon Assimilation
Source: Microb Ecol. 2023 Jul 22;86(4):2642–54. doi: 10.1007/s00248-023-02271-1 (PMC10640455; doi:10.1007/s00248-023-02271-1)
Supplement: Supplementary file 1 — (DOCX 1480 kb) [file 248_2023_2271_MOESM1_ESM.docx]

Supplementary material

Tributary inflows to a regulated river influence bacterial communities and increase bacterial carbon assimilation

Microbial Ecology

Author INFORMATION

|  | ORCID |
| --- | --- |
| Lauren O’Brien^1^* | 0000-0002-1504-6613 |
| Nachshon Siboni^2^ | 0000-0001-6082-0949 |
| Justin R. Seymour^2^ | 0000-0002-3745-6541 |
| Matthew Balzer^1^ | 0000-0003-1689-0506 |
| Simon Mitrovic^1^ | 0000-0002-5528-2215 |

^1^ School of Life Sciences, University of Technology Sydney, Ultimo, NSW, Australia

^2^ Climate Change Cluster, University of Technology Sydney, Ultimo, NSW, Australia

* lauren.e.obrien@student.uts.edu.au

(a)


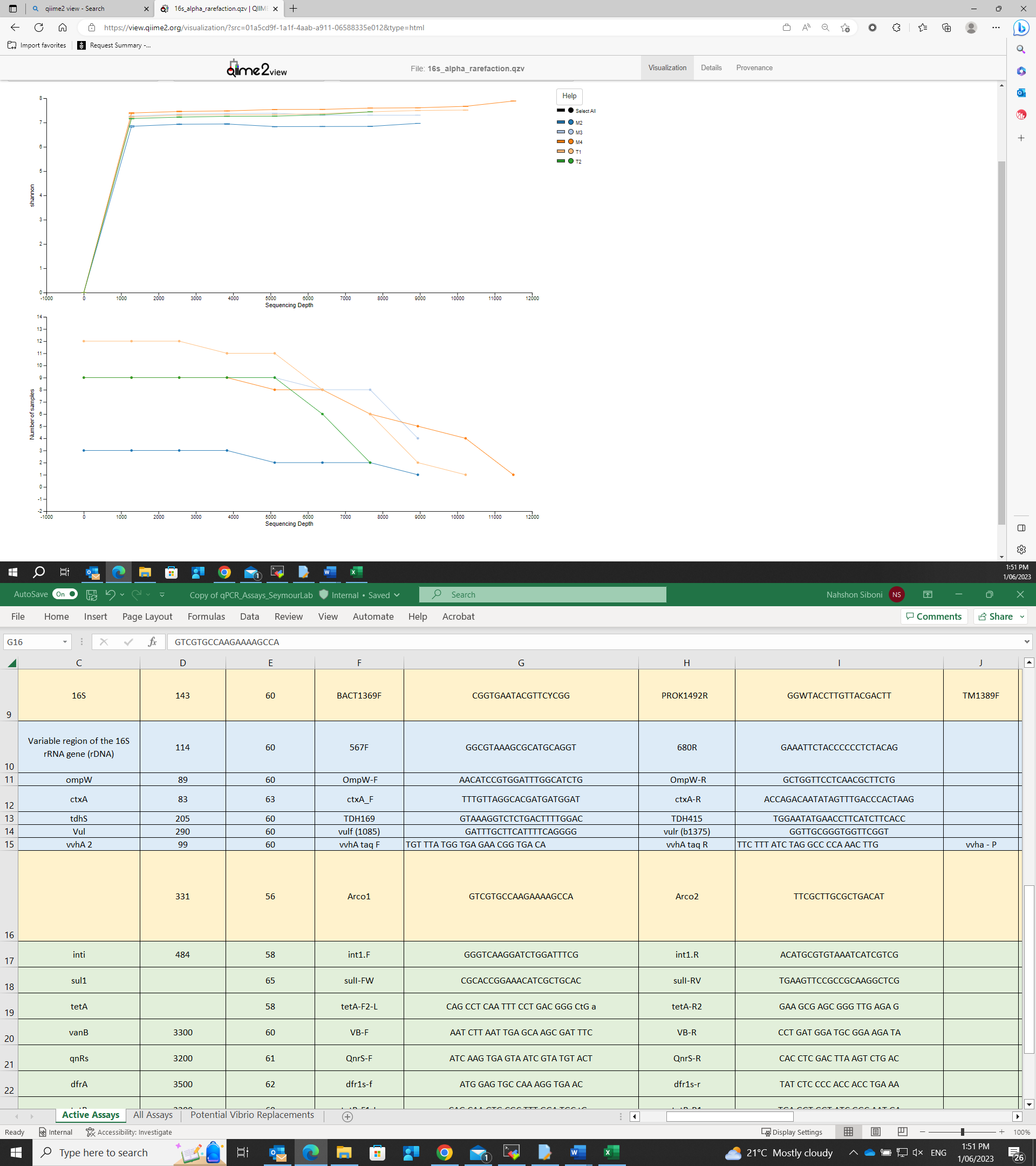


(b)


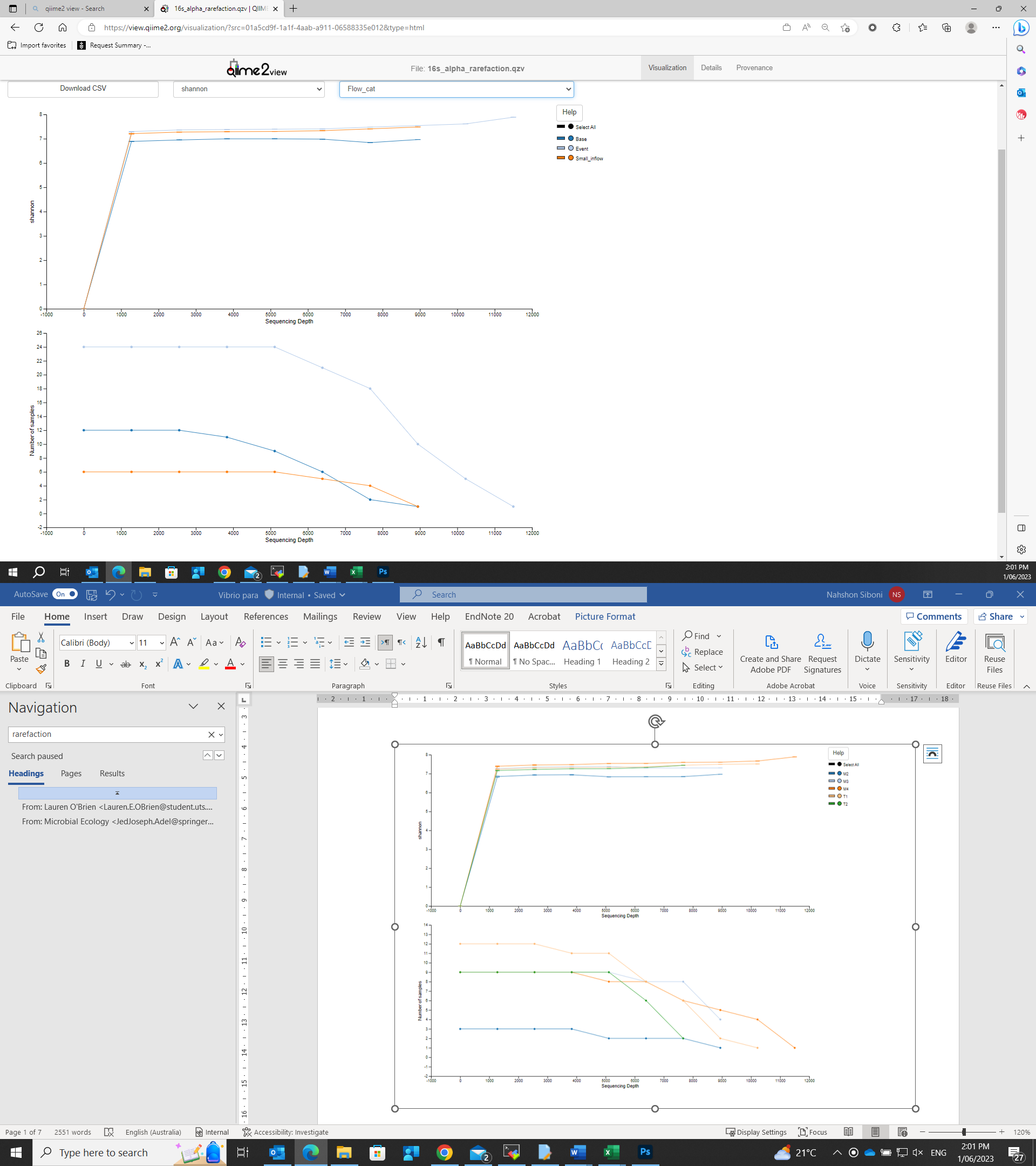


(c)


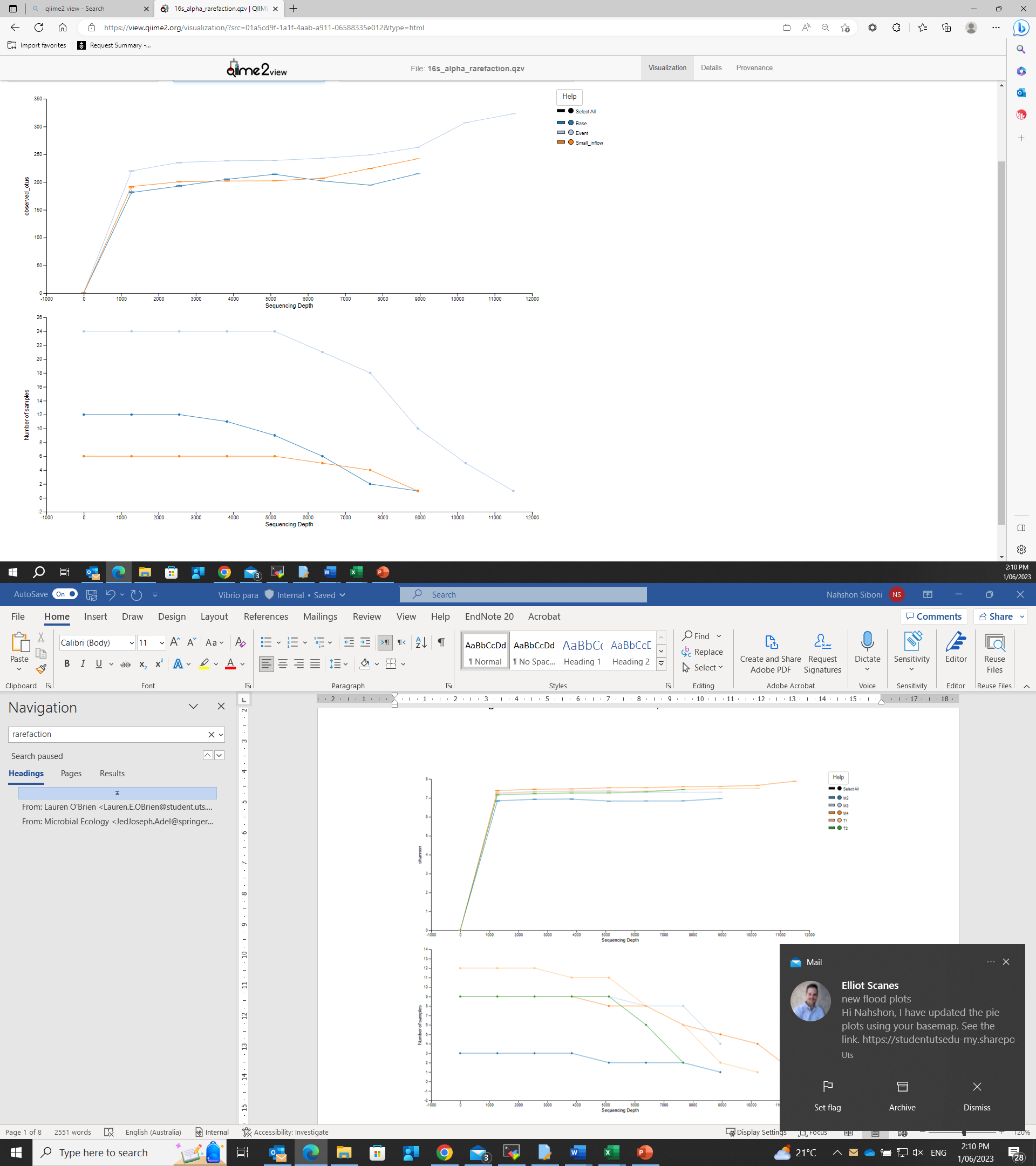


(d)


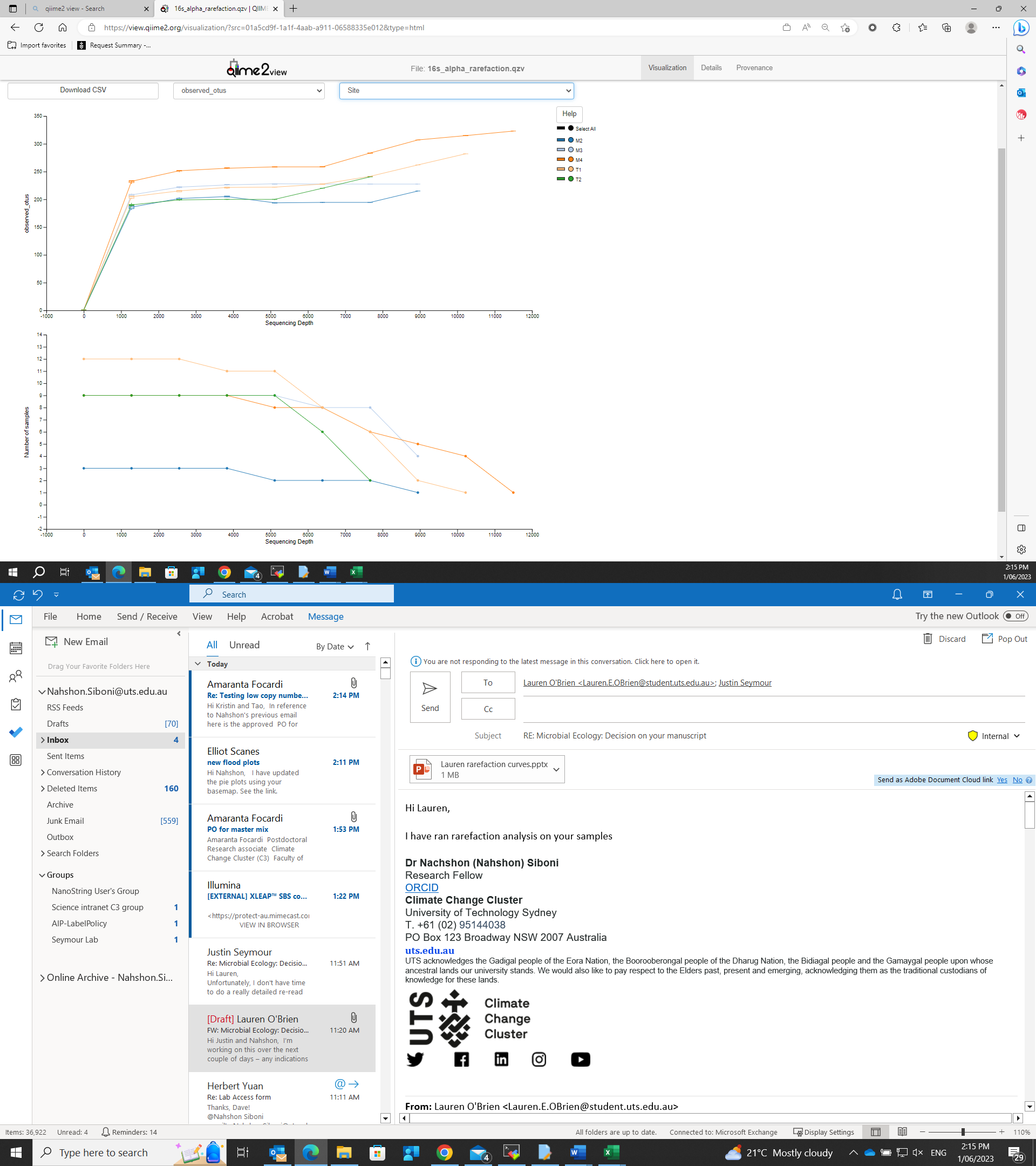


**Supplementary figure S1.** Rarefaction curves used to justify rarefaction to 3530 reads per sample for ASVs sequenced from five sites in the Lachlan River catchment during a tributary driven flow pulse. Plots are grouped by (a) Site x Shannon diversity index, (b) Flow category x Shannon diversity index, (c) Flow category x observed ASVs, and (d) Site x observed ASVs. Note that in plot (d) some samples at site M4 have more than 3530 observed ASVs, however the Shannon diversity index (a) was unchanged above this cut off.

**Supplementary table S3.** Volumetric bacterial carbon production (BCP*_vol_*) values for incubations conducted with and without an amendment of Lindstrom’s L16 growth media made without carbon [42].

| **ID** | **BCP*_vol_* with no amendment (µg C L^-1^ hr^-1^)** | **BCP*_vol_* with L16 amendment (µg C L^-1^ hr^-1^)** |
| --- | --- | --- |
| M1-1 | 4.95 ± 4.9 | 4.94 ± 4.9 |
| M1-2 | 2.97 ± 3 | 2.84 ± 2.8 |
| M1-3 | 2.44 ± 2.4 | 2.23 ± 2.2 |
| M1-5 | 2.03 ± 2 | 2.56 ± 2.6 |
| M2-1 | 4.06 ± 4.1 | 4.02 ± 4 |
| M2-2 | 3.09 ± 3.1 | 3.08 ± 3.1 |
| M2-3 | 2.11 ± 2.1 | 2.1 ± 2.1 |
| M2-4 | 1.96 ± 2 | 1.99 ± 2 |
| M2-5 | 2.33 ± 2.3 | 2.3 ± 2.3 |
| M3-1 | 3.54 ± 3.5 | 3.6 ± 3.6 |
| M3-2 | 4.4 ± 4.4 | 4.34 ± 4.3 |
| M3-3 | 1.69 ± 1.7 | 1.59 ± 1.6 |
| M3-4 | 1.55 ± 1.5 | 1.51 ± 1.5 |
| M3-5 | 2.56 ± 2.6 | 2.57 ± 2.6 |
| M4-2 | 2.72 ± 2.7 | 2.55 ± 2.6 |
| M4-4 | 1.11 ± 1.1 | 1.11 ± 1.1 |
| M4-5 | 2 ± 2 | 1.86 ± 1.9 |
| T1-1 | 3.47 ± 3.5 | 3.35 ± 3.4 |
| T1-2 | 6.18 ± 6.2 | 5.93 ± 5.9 |
| T1-3 | 3.61 ± 3.6 | 3.36 ± 3.4 |
| T1-5 | 2.18 ± 2.2 | 2.01 ± 2 |
| T2-1 | 1.66 ± 1.7 | 1.66 ± 1.7 |
| T2-2 | 2.62 ± 2.6 | 2.53 ± 2.5 |
| T2-4 | 1.14 ± 1.1 | 1.1 ± 1.1 |
| T2-5 | 2.04 ± 2 | 1.89 ± 1.9 |

#

**Supplementary figure S2.** SIMPROF dendrogram indicating similarity of bacterial community composition based on 16S rRNA sequencing from five sites in the Lachlan River catchment during a tributary driven flow pulse. Sample names indicate sample site and day of sampling – event flow occurred on days 4-5. Significant clusters are identically coloured.

**Supplementary table S4.** Bacterial amplicon sequence variants (ASVs) that contributed most to the difference between flow categories during a tributary driven flow pulse on the Lachlan River as identified by SIMPER analysis. Significant contributors (α < 0.05) in the cumulative top 10% are shown. ASVs that increased during event flow are highlighted with italic text.

| ASV ID | Proportion of contribution | Order | Family | Genus | Event flow mean relative abundance | Base/small inflow mean relative abundance |
| --- | --- | --- | --- | --- | --- | --- |
| **Base flow vs event flow** | | | | | | |
| ASV_1 | 0.00825 | Cytophagales | Spirosomaceae | Pseudarcicella | 21.6 | 53.9 |
| ASV_2 | 0.00748 | Cytophagales | Spirosomaceae | Pseudarcicella | 20.6 | 54.2 |
| ASV_7 | 0.00670 | Burkholderiales | Burkholderiaceae | Polynucleobacter | 17.5 | 45.4 |
| ASV_9 | 0.00588 | Cytophagales | Spirosomaceae | Pseudarcicella | 18.2 | 42.5 |
| ASV_12 | 0.00587 | Burkholderiales | Burkholderiaceae | Polynucleobacter | 15.7 | 40.9 |
| ASV_30 | 0.00585 | Burkholderiales | Alcaligenaceae | GKS98 freshwater group | 7.10 | 36.2 |
| ASV_32 | 0.00544 | Burkholderiales | Alcaligenaceae | GKS98 freshwater group | 7.05 | 34.2 |
| *ASV_4* | *0.00523* | *Burkholderiales* | *Burkholderiaceae* | *Polynucleobacter* | *45.7* | *21.1* |
| ASV_38 | 0.00507 | Micrococcales | Microbacteriaceae | Rhodoluna | 6.57 | 32.1 |
| ASV_37 | 0.00504 | Micrococcales | Microbacteriaceae | Rhodoluna | 7.57 | 31.1 |
| ASV_23 | 0.00490 | Burkholderiales | Alcaligenaceae | GKS98 freshwater group | 18.6 | 31.2 |
| *ASV_5* | *0.00489* | *Cytophagales* | *Spirosomaceae* | *Pseudarcicella* | *44.3* | *21.8* |
| *ASV_10* | *0.00474* | *Cytophagales* | *Spirosomaceae* | *Pseudarcicella* | *40.8* | *18.7* |
| **Small inflow vs event flow** | | | | | | |
| ASV_1 | 0.0104 | Cytophagales | Spirosomaceae | Pseudarcicella | 68.5 | 21.6 |
| ASV_11 | 0.00924 | Burkholderiales | Burkholderiaceae | Polynucleobacter | 61.8 | 19.0 |
| ASV_2 | 0.00908 | Cytophagales | Spirosomaceae | Pseudarcicella | 63.0 | 20.6 |
| ASV_7 | 0.00871 | Burkholderiales | Burkholderiaceae | Polynucleobacter | 57.7 | 17.5 |
| ASV_6 | 0.00838 | Burkholderiales | Burkholderiaceae | Polynucleobacter | 57.0 | 18.5 |
| ASV_3 | 0.00830 | Burkholderiales | Burkholderiaceae | Polynucleobacter | 59.5 | 21.9 |
| ASV_13 | 0.00746 | Cytophagales | Spirosomaceae | Pseudarcicella | 50.2 | 16.0 |
| ASV_12 | 0.00723 | Burkholderiales | Burkholderiaceae | Polynucleobacter | 49.3 | 15.7 |

**Supplementary table S5.** Bacterial amplicon sequence variants (ASVs) that contributed most to the difference between tributaries T1 and T2 during a flow pulse on the Lachlan River as identified by SIMPER analysis. Significant contributors (α < 0.05) in the cumulative top 10% are shown.

| ASV ID | Proportion of contribution | Order | Family | Genus | T1 mean relative abundance | T2 mean relative abundance |
| --- | --- | --- | --- | --- | --- | --- |
| ASV_23 | 0.00735 | Burkholderiales | Alcaligenaceae | GKS98 freshwater group | 8.67 | 48.3 |
| ASV_25 | 0.00681 | Burkholderiales | Alcaligenaceae | GKS98 freshwater group | 7.25 | 44.2 |
| ASV_4 | 0.00577 | Burkholderiales | Burkholderiaceae | Polynucleobacter | 27.9 | 45.1 |
| ASV_31 | 0.00563 | Burkholderiales | Alcaligenaceae | GKS98 freshwater group | 7.33 | 37.1 |
| ASV_5 | 0.00551 | Cytophagales | Spirosomaceae | Pseudarcicella | 25.4 | 43.7 |
| ASV_10 | 0.00537 | Cytophagales | Spirosomaceae | Pseudarcicella | 24.0 | 42.8 |
| ASV_39 | 0.00529 | Micrococcales | Microbacteriaceae | *unassigned* | 5.58 | 34.2 |
| ASV_8 | 0.00498 | Cytophagales | Spirosomaceae | Pseudarcicella | 23.3 | 39.9 |
| ASV_35 | 0.00479 | Micrococcales | Microbacteriaceae | Rhodoluna | 6.33 | 31.9 |
| ASV_40 | 0.00473 | Micrococcales | Microbacteriaceae | *unassigned* | 5.58 | 31.1 |
| ASV_15 | 0.00466 | Burkholderiales | Burkholderiaceae | Polynucleobacter | 20.4 | 37.1 |
| ASV_16 | 0.00454 | Burkholderiales | Burkholderiaceae | Polynucleobacter | 21.5 | 38.1 |
| ASV_17 | 0.00432 | Cytophagales | Spirosomaceae | Pseudarcicella | 20.2 | 35.3 |
